# B2-NiAl Strengthened PH 13–8 Mo Steel Under Fe^+^ Ion Irradiation: Precipitate Stability and Interaction with Damage Defects

**DOI:** 10.3390/ma18245524

**Published:** 2025-12-09

**Authors:** Zijing Huang, Roudi Yang, Ming Chen, Yiting Ding, Chenglu Liu, Jiahan Zhang, Binghang Li, Ce Zheng

**Affiliations:** 1 College of Energy, Xiamen University, Xiamen 361005, China; roudiyang@163.com (R.Y.); 13560897007@163.com (M.C.); dyt18091612112@163.com (Y.D.); lilia_liuchenglu@163.com (C.L.); zhangjiahan666@outlook.com (J.Z.); binghangli47@gmail.com (B.L.); 2 Science and Technology on Thermo Structural Composite Materials Laboratory, Northwestern Polytechnical University, Xi’an 710072, China; cezheng@nwpu.edu.cn

**Keywords:** PH 13–8 Mo steel, B2 NiAl precipitate, ion irradiation, irradiation stability, dislocations

## Abstract

**Highlights:**

**What are the main findings?**

- In our work, we find that NiAl precipitates larger than 5nm will survived after 8dpa irradiation at room temperature;
- We find that Moiré fringes are highly related with the precipitates which lose their superlattice crystal structure.

**What are the implications of the main findings?**

- Radiation-induced precipitate disordering is the main reason for the irradiation dissolution phenomenon.
- The Moiré fringes formation is attributed to the twisted effect between precipitates’ low index crystal planes overlapped with the BCC matrix at various angles.

**Abstract:**

To investigate the radiation stability of the intermetallic in PH 13–8 Mo steel, precipitates with different sizes were generated and then the samples are irradiated with 400 keV Fe^+^ at room temperature with maximum damage up to 8 dpa. The pre- and post-irradiation samples are examined with selected area electron diffraction (SAED), scanning transmission electron microscopy (STEM) and Energy Dispersive Spectroscopy (EDS). Before the irradiation, B2 NiAl precipitates are uniformly distributed in matrix with increased sizes of 2.5, 4.9 and 8.1 nm. After the irradiation, the intensity of SAED superlattice pattern of B2 NiAl with 8.1 nm diminishes rather than disappeared in the remaining samples, indicating that the ordered B2 structure of NiAl precipitates of smaller size are mostly destroyed. EDS results proves that no elemental diffusion took place between the precipitates and matrix. Moiré fringes are found to be located beside dissolved precipitates attributed to radiation-enhanced diffusion. This work will provide advice for the material design of other intermetallic strengthened alloys especially in nuclear applications.

## 1. Introduction

Martensitic aging alloys are low-carbon, high-strength steels suitable for key components in industries such as the aircraft and aerospace industries (e.g., landing gear, rocket motor casings and so on) and essential for medical equipment in the dental filed [1]. In the International Thermonuclear Experimental Reactor (ITER) project, the selected material would have to suffer a critical neutron irradiation environment which might lead to serious material degradation [2]. The martensitic aging alloy PH 13–8 Mo can generate specific nano-sized second phase particles within the material matrix through appropriate heat treatment processes [3,4,5,6,7,8,9,10], and the dispersed nano-sized second phase particles typically enhance the high-temperature mechanical properties of the material, especially the high-temperature creep resistance [11,12,13,14]. Therefore, martensitic aging steel has become a promising candidate material for the first wall bolts of the fusion reactor of in-vessel structures [14,15].

In a high neutron flux real service environment, the materials would accumulate irradiation damage continuously, which would eventually degrade the mechanical properties. The numerous fine and dispersed precipitates induced by the aging process in martensitic aging steel would provide extra surface to absorb defects, which would enhance the material’s irradiation resistance [15,16,17]. Grey et al. from the U.S. Naval Laboratory initiated irradiation studies on martensitic aging steel as early as 1970 [12]. The inception of the U.S. government’s Generation IV reactor program in 2006 significantly propelled the research and development of novel reactor materials. Subsequent studies on the irradiation characteristics of second-phase nanoparticles revealed that the complete dissolution of these particles could deteriorate high-temperature creep resistance, diminish the material’s ultimate yield strength, and potentially trigger stress corrosion cracking during irradiation, leading to material deterioration and failure [18]. Since the nano-sized particles in martensitic aging steel played a key role in studying the irradiation resistance of materials, a detailed investigation on the precipitate morphology in response to irradiation could serve as a foundation for designing other nano-particle-strengthened alloys (e.g., ODS steel, nickel-based superalloys, particle-reinforced tungsten-based alloys).

Van et al. examined the B2 phase stability in the martensitic aging alloy PH 13–8 Mo under neutron irradiation at 300 °C [5]. Their findings indicated that after being irradiated at 0.5, 1 and 2 dpa, analysis via transmission electron microscopy (TEM) revealed the persistence of the original NiAl phase, approximately 8 nm in size, with no alteration in size or number density attributed to neutron irradiation, suggesting the stability of NiAl under irradiation [18,19,20]. Hofer et al. similarly observed constant radius and number density of the NiAl phase pre- and post-proton irradiation at room temperature up to approximately 2 dpa in PH 13–8 Mo, employing atom probe tomography (APT). Both results showed that the composition of the precipitates changed after irradiation [21]. Zheng’s results and analysis revealed a decrease in Al concentration and an increase in Si concentration in the precipitates, indicating irradiation-induced alloy element redistribution [10]. However, there was no evidence for the stability of NiAl precipitates in PH 13–8 Mo under high-dose irradiation, particularly the crystallographic stability as well as destabilization mechanisms. A further investigation focused on the precipitate morphology and its evolution after irradiation would be necessary. This study examines the evolution of the B2 NiAl phase, a secondary phase particle in PH 13–8 Mo martensitic aging steel, in pre- and post-irradiation status. This work offers an experimental foundation for comprehensive understanding of the interplay between irradiation and secondary phase particles.

## 2. Materials and Methods

The actual type PH 13–8 Mo steel used in the experiment was purchased from Bohler-Uddehlm (Uddehlm, Beijing, China). The alloy was analyzed with a SPECTROBLUE FMX36 (SPECTRO, Kleve, Genmany) analysis instrument which revealed the nominal composition of alloy as shown in Table 1. The flake samples with 10 mm × 10 mm × 1 mm were thermally aged in a GSL 1500X-50 (Shenzhen Kejing Star Technology Company, Shenzhen, China) muffle furnace with precise temperature control (±1 °C) at 723 K, 748 K and 773 K for 20 h, respectively, then cooled with the furnace. Subsequently, the aged samples were mechanically polished with SiC sandpapers to prepare the foils with thickness less than 100 μm. A dedicated punching machine was used to conduct 3 mm disks and then the disks were further polished down to 50 μm. In the last step, the disks were twin-jetted with electrolyte of 5% perchloric acid ethanol solution.

**Figure 1:**
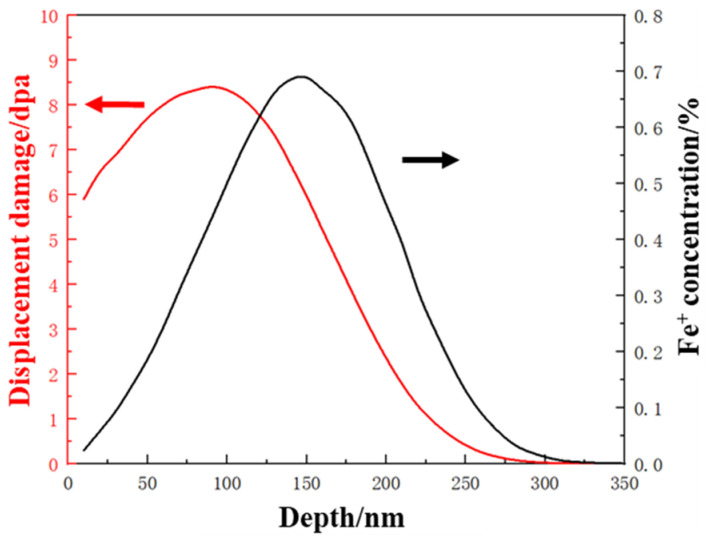
SRIM calculated damage profiles generated by implanted 400 keV Fe^+^ ions in PH 13–8 Mo.

Prepared TEM foils with central holes were examined in TEM prior to an ex situ irradiation experiment. The irradiation experiments were carried out at room temperature with 400 keV Fe^+^ ions in an NEC implanter chamber in the College of Energy at Xiamen University. The irradiation flux was selected as 1.85 × 10^12^ ions cm^−2^s^−1^ and the total fluence was 3.33 × 10^15^ ions cm^−2^. SRIM 2008 (stopping and range of ions in matter) is a commonly used software in ion irradiation research for simulating the impact of ions on matter. In this case, a well accepted and recommended Kinchin-Pease model was selected [22]. The Ed of the main elements like Fe, Cr and Ni were set as 40 eV while 25 eV for Al and 60 eV for Mo. With a total fluence of 3.33 × 101^5^ ions cm^−2^ at a depth of ~100 nm, an equivalent dose of 8.2 dpa was estimated. As shown in Figure 1, the black curve explained the radiation damage induced by the 400 keV Fe^+^ ions implanted into PH 13–8 Mo, and the red curve was the implanted Fe^+^ concentration.

Conventional and high-resolution TEM (HRTEM) were performed with a Thermofisher TECNAI G2 F30 TWIN TEM (Thermo Fisher, Waltham, MA, USA). Scanning transmission electron microscope (STEM) and energy dispersive spectroscopy (EDS) was used to evaluate the morphology and element segregation of precipitates. ImageJ 1.54g was used to measure the size and count the number density of precipitates and dislocations from dark field (DF) images and bright field (BF) TEM images.

## 3. Results and Discussion

### 3.1. Characterization of the Precipitated Thermal Aging Samples

To avoid overloading the paper, samples were numbered with #1, #2 and #3 according to the aging temperature from low to high, as shown in Table 2.

**Figure 2:**
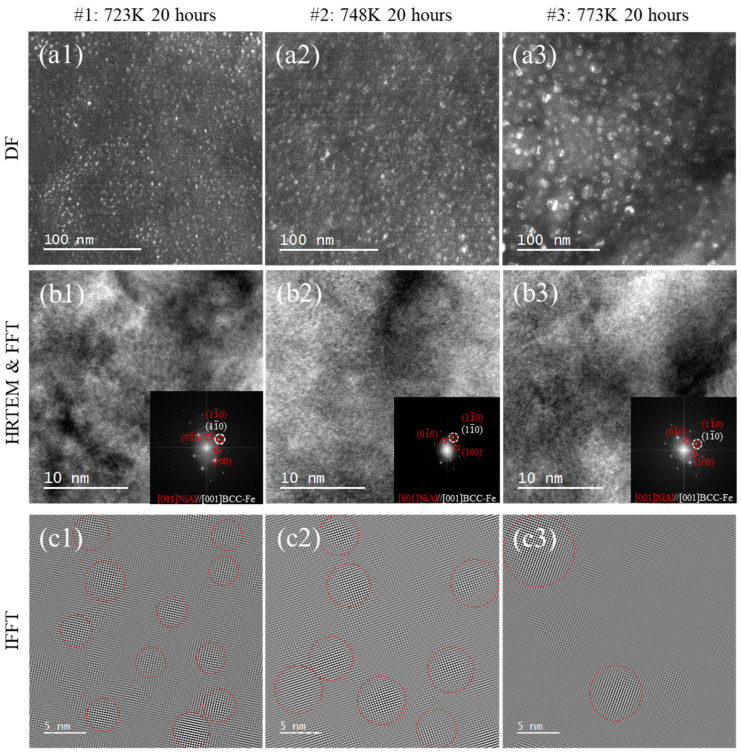
Microstructure of aged samples before irradiation: (**a1**–**a3**) DF of samples aged 20 h at 723 K, 748 K and 773 K; (**b1**–**b3**) HRTEM and FFT patterns of samples aged 20 h at 723 K, 748 K and 773 K; (**c1**–**c3**) IFFT of samples aged 20 h at 723 K, 748 K and 773 K.

Figure 2(a1–a3) shows the DF TEM images of the (001) NiAl superlattice before irradiation and the number density of NiAl estimated. The diffraction spots of the DF images as shown in Figure 2(a1,b1,c1) were from the unirradiated state of the samples. Fine uniformly distributed spherical NiAl precipitates were formed in the matrix and attributed to the thermal aging effect. The thickness of the transmission electron microscope foil was estimated by the CBED method [23], the area density of the NiAl phase was converted as number density as well as those applied in the work of Nie and Kong et al. [24,25]. The number densities of the precipitates in #1, #2 and #3 are 2.01 ± 0.16 × 10^22^ m^−3^, 9.4 ± 0.15 × 10^21^ m^−3^ and 3.5 ± 0.04 × 10^21^ m^−3^ (Table 2), respectively. As shown in Figure 2(b1–b3), the spots marked with red circles in the FFT images were identified as the B2 NiAl precipitates. After selecting those superlattice spots in FFT and inversing FFT spots, image contrast of the precipitated phases corresponding to the superlattice were enhanced as shown in Figure 2(a3–c3). The precipitated NiAl in #1–#3 were marked with red dot circles in Figure 2(c1–c3). The statistical results of NiAl redius were shown in Table 2 indicating the average NiAl sizes in #1, #2 and #3 were 2.5 ± 0.3 nm, 4.9 ± 0.3 nm and 8.1 ± 1.3 nm, respectively.

### 3.2. Phase Stability of NiAl Under Irradiation

**Figure 3:**
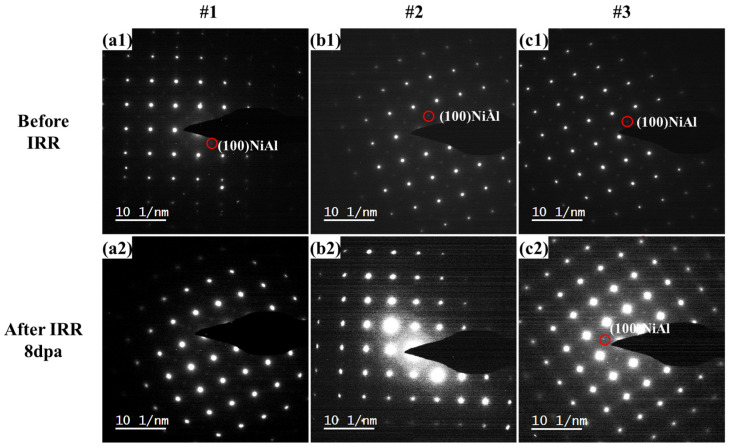
Comparison of selected area electron diffraction (SAED) images before and after irradiation: before irradiation (**a1**,**b1**,**c1**); after Fe^+^ ions irradiated 8 dpa (**a2**,**b2**,**c2**).

The SAED images of #1, #2 and #3 pre- and post-irradiation were shown in Figure 3. NiAl superlattice reflections were marked with red circles as shown in Figure 3. After 8 dpa irradiation, the NiAl superlattice reflections almost disappeared in samples #1 and #2, suggesting the smaller NiAl precipitates (~2.5 and ~4.9 nm) could have been damaged during the irradiation. However, it is interesting to find the superlattice spots in the SAED result (Figure 3(c2)) for the #3 sample, even though the superlattice diffraction pattern was much weaker, indicating some of the 8.1 nm NiAl precipitates could have been ruined while the rest survived from the irradiation.

**Figure 4:**
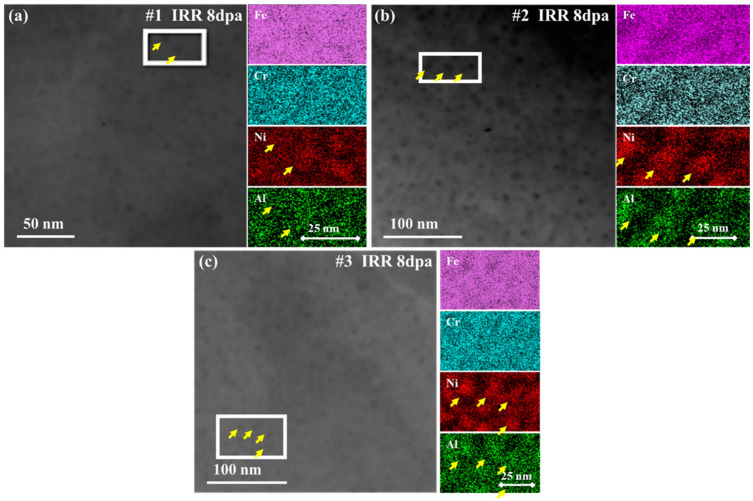
STEM and EDS of irradiated samples, 8 dpa. (**a**) #1; (**b**) #2; (**c**) #3.

Figure 4 shows the STEM and EDS results of #1, #2 and #3 after irradiation and the yellow arrows marked the precipitates.

According to the research results of C. Zheng et al., after aging NiAl in the sample appears as dark spherical particles in the STEM image [10]. In this study, the dark particles could still be observed in the samples after irradiation. The right side of the STEM images is the EDS results corresponding to the red frame. The results show that the dark particles in each sample are enriched in Ni and Al elements. It is worth noticing that no elemental dispersion was observed in the three types of samples, and the spinodal decomposition characteristics were not obvious. But when studying the δ-ferrites of 308 ASSW after proton irradiation, Byeong Seo Kong et al. detected the formation of nickel-rich and silicon-rich clusters in the δ-ferrite of spinodal decomposition, and spinodal decomposition with an average wavelength of 9 nm [25]. However, in this study, the statistical results show that the wavelengths of spinodal decomposition are all around 0.5 nm. In addition, the size of Moiré fringe regions induced by irradiation were slightly larger than the size of the original precipitate, indicating that when the radiation damage dose is 8 dpa, the spinodal decomposition process in the sample has not completely ended, so no obvious element separation and diffusion is observed.

**Figure 5:**
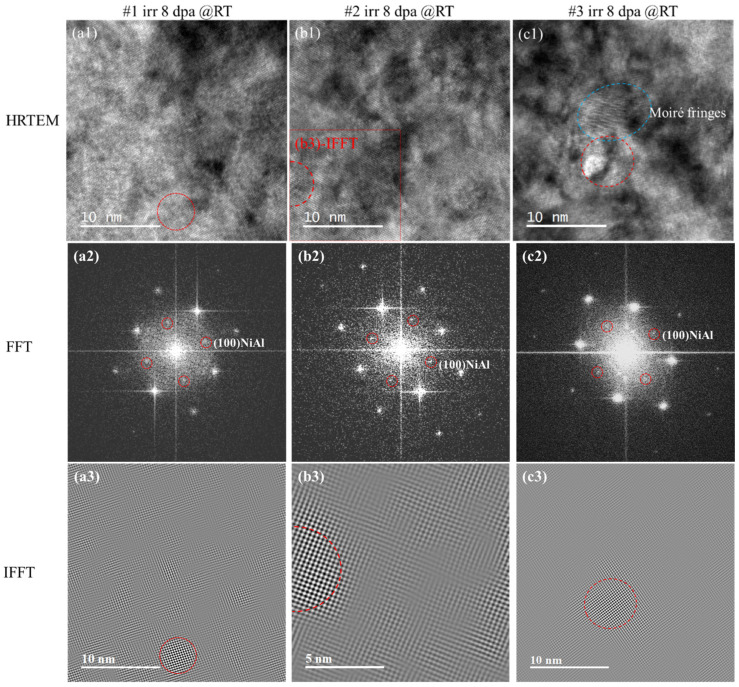
HRTEM images, FFT images and corresponding IFFT images after irradiated 8 dpa: (**a1**–**a3**) #1; (**b1**–**b3**) #2; (**c1**–**c3**) #3. The red circles in the FFT images are the NiAl superstructure spots. The red circles in the IFFT images represent NiAl, and the blue represent Moiré fringes.

Figure 5 shows the HRTEM, FFT and corresponding IFFT images of #1, #2 and #3 after irradiation. FFT of the HRTEM in both #1 and #2 samples were consistent with the SAED results indicating disappearance of NiAl precipitates in most examined areas after irradiation. In our work, further examination was conducted with Digital Micrograph (DM) software (v3.30.2016.0), using a small ROI frame binned with FFT in the HRTEM graphs. Coherent superlattice diffractions spots shown in Figure 5(a2) associated with the B2 precipitate structure were recognized at a very rare rate. The related precipitate survived from the irradiation was marked with red dash-line circle in the #1 HRTEM images Figure 5(a1). Same as in #2 HRTEM (Figure 5(b1,b2)). The specified NiAl precipitates in #1 and #2 were reconducted into IFFT images (Figure 5(a3,b3)) marked with red circles. The sizes of post-irradiation residual NiAls found in HRTEM were consistent with the pre-irradiation data. However, since the residuals were so rare, it was not likely to derive the average size and number density in this work. In sample #3, the FFT of HRTEM images could easily derive residual NiAls information as shown in Figure 5(c1) and Figure 5(c2). The related IFFT was conducted in Figure 5(c3).

Afterwards, the SAED superstructure diffraction spots of NiAl in #1 and #2 were lost while FFT revealed a few surviving precipitates after irradiation; for #3, those super spots deteriorated but remained present in both SAED and FFT. Such phenomena indicated that after the irradiation damage accumulated up to 8 dpa, most of NiAl precipitates with size <5 nm were destroyed, and thus the volume density of residual precipitates was too low to provide contrast in SAED. Precipitates with size >8 nm deteriorated with irradiation but showed more irradiation tolerance than the smaller ones in #1 and #2.

Before the irradiation, the B2 NiAl precipitates appeared homogeneously distributed in the BCC matrix in #1–#3 samples. Since the Ni atom occupied the eight corner sites while the Al took the (1/2, 1/2, 1/2) site, the SAED provided sharp superlattice patterns. It is interesting to find that precipitates with sizes less than 5 nm merely survived after irradiation while the larger-sized ones in #3 mostly remained. The size of the precipitated phase is an important factor affecting the stability of the second phase by ballistic mixing. The study on Cr-rich α′ precipitates could be a good example. The FeCr binary alloy serves as a representative model alloy in which small α′ precipitates can be generated through controlled methods [17,26,27,28]. To examine the ballistic dissolution of these small precipitates, Vörtler et al. utilized molecular dynamics (MD) simulations to replicate the cascade irradiation damage produced by 20 keV primary knock-on atoms (PKA). Their study demonstrated that in FeCr alloys with varying chromium contents (5, 10 and 15 wt.%), the 5 nm α′ precipitates remained undissolved and unaltered [29,30]. Furthermore, Tikhonchev et al. [31,32] noted that chromium-rich clusters ranging from 1 to 5 nm in size exhibited re-dissolution, particularly those measuring 1 to 2 nm, under similar conditions. Additionally, Harrison et al. used MD methods to simulate the dissolution mechanism of the Fe-Cu system and found that the smaller-sized precipitate has a larger surface-to-volume ratio, and compared with the larger precipitates, it has a greater relative reactivity solubility [33,34,35]. In the current case, the disappearance of superlattice SAED pattern indicated that smaller B2 NiAl precipitates lost their ordered crystal structure attributed to atom displacements created within collision cascades. In such precipitates, dissolution process solute atoms were ballistically driven into the surrounding matrix under control by recoil dissolution or disordering dissolution. Under the condition of either low temperatures or high irradiation fluxes, both mechanisms contribute significantly to the increase in the solute solubility limitation, but the ballistic mixing mechanism is much more efficient. As mentioned above, #1–#3 samples were irradiated at the same time indicating that displacement rates and temperature played no roles, and the sink strength of precipitates dominated the whole recoil dissolution process. In other words, 8.1 nm is the critical size of B2 NiAl precipitates as it consequently provided the most sink strength for solute atoms to maintain a kinetic equilibrium concentration between the precipitates and matrix. In full consideration, the competition between radiation-induced disordering and thermal reordering should not be ignored. Since the system thermal status at room temperature (<0.2 T_m_) retained the mobility of radiation vacancies, the thermal effect could only be brought on by a cascade thermal spike which was far from enough for the thermal reordering.

### 3.3. Irradiation-Induced Moiré Fringes

**Figure 6:**
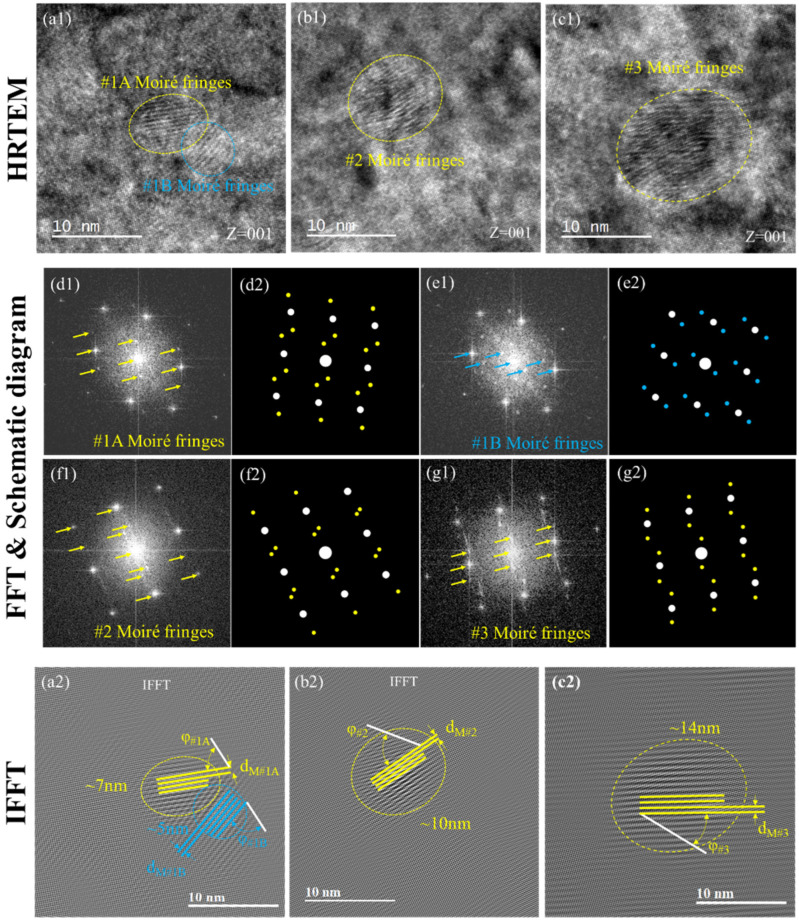
HRTEM, FFT and IFFT of the Moiré fringes of #1, #2 and #3 after 8 dpa: (**a**–**c**) HRTEM of #1, #2 and #3; (**d1**,**d2**) FFT and the schematic diagram of yellow Moiré fringe in (**a**); (**g1**,**g2**) FFT and the schematic diagram of blue Moiré fringe in (**a**); for #2 (**b**,**e1**,**e2**); for #3 (**c**,**f1**,**f2**).

Moiré fringes were generally found in the HRTEM images of post-irradiation samples while B = [001], as shown in Figure 6(a1–c1). Conduct FFT on the domains of both Moiré fringes (yellow and blue dotted circles) in Figure 6(a1) resulted in two sets of spotted patterns as shown in Figure 6(d1,e1), and it should be noticed that the sideband spots were found on both sides of the diffraction spots. FFT schematics were made according to the relative distance and direction of the sideband spots and the main spot in Figure 6(d2,e2), where the white dot represents the main spot and the colored dot represents the sideband spots; so were the Moiré regions in #2 and #3 as shown in Figure 6(f1–g2). Sideband spots appeared in FFT images which indicated that there were micro regions bearing stress with the same crystal structure in the solid solution matrix. Since the size of the Moiré regions of #1, #2 and #3 after irradiation is relatively consistent with the size of B2 NiAl, it would be reasonable to attribute the Moiré pattern to the radiation-induced displacement areas beside the NiAl precipitate. In Tu’s work [36], a nano-Moiré pattern analysis method was introduced to reveal the formation of Moiré fringes. Based on the understanding that the Moiré fringes were related to the overlapping of one group of equispaced parallel lattice over another, the distinct spacing of the Moiré pattern d_M_ and its orientation angle overlapping with the matrix *φ* can be calculated with Equations (1) and (2).

(1) $$d_{M}=\frac{d_{0}d_{1}}{\sqrt{d_{1}^{2}+d_{0}^{2}-2d_{0}d_{1}\cos\alpha }}$$

(2) $$\sin\varphi =\frac{d_{0}\sin\alpha }{\sqrt{d_{1}^{2}+d_{0}^{2}-2d_{0}d_{1}\cos\alpha }}$$

where *d*_0_ is the lattice spacing of the matrix *d_F/M_*_(110)_, *d*_1_ is the altered spacing of B2 NiAl precipitates *d_NiAl_*_(*hkl*)_, *d_M_* is the spacing of the moiré pattern, and *α* is the overlapping angle of the NiAl group (hkl) with respect to the F/M matrix group (110). The conducted IFFT process on the HTREM images to obtain more precise Moiré patterns as well as the background atomic images as shown in Figure 6(a2–c2), where the white lines were the (110) plane of the matrix, and the yellow and blue lines were the Moiré pattern spacings. With the measured *d_F/M_*_(110)_, *d_M_* and *φ*, *d_NiAl_*_(*hkl*)_ and *α* could be solved with Equations (1) and (2) as summarized in Table 3.

As NiAl precipitates were coherent with the matrix, the lattice was constant in domains where either the survived or recoiled dissolution NiAl precipitates would not change much in number after the irradiation ($a_{0(F/M)}\approx a_{0(NiAl)}$), which means the square ratio of *d*_0_/*d*_1_ would be mainly determined by the Miller index of related crystal planes. Apply the d-spacing expression of the BCC structure in the term of $\frac{d_{0}^{2}}{d_{1}^{2}}$, such term could be rewritten as the following,

(3) $$h+k+l=odd\;number\;\frac{d_{\left(100\right)}^{2}}{d_{\left(hkl\right)}^{2}}=\frac{a_{0\left(\frac{F}{M}\right)}^{2}(h^{2}+k^{2}+l^{2})}{a_{0\left(NiAl\right)}^{2}(1^{2}+1^{2}+0^{2})}=h^{2}+k^{2}+l^{2}$$

(4) $$h+k+l=even\;number\;\frac{d_{\left(100\right)}^{2}}{d_{\left(hkl\right)}^{2}}=\frac{a_{0\left(\frac{F}{M}\right)}^{2}(h^{2}+k^{2}+l^{2})}{a_{0\left(NiAl\right)}^{2}(1^{2}+1^{2}+0^{2})}=\frac{\left(h^{2}+k^{2}+l^{2}\right)}{4}$$

Subsequently, the calculated $\frac{d_{0}^{2}}{d_{1}^{2}}$ would be rounded up to an integer for further crystal plane calculation. The results were shown in Table 4, indicating the possible overlapping plane family (*d*_1_) over the F/M matrix could be identified as shown in Table 2. It is interesting to find that for the selected Moiré patterns in all three samples, the solutions from Table 3 shared close values in both α and the crystal plane of d_1_. The results indicated that the observed Moiré fringes could be but not necessarily resulted from twisted {100} planes with ~30° or {110}, {111} with smaller angles.

As discussed before, the radiation-induced vacancy diffusion was restrained and the smaller B2 NiAl lost their ordered structure, which is mostly attributable to their weak sink strength.

Without thermal reordering, the short-range atomic redistribution returned solute atoms back randomly to the lattice sites which increased the lattice distortion in comparison with the unirradiated state of regions where B2 NiAl is located. Moreover, as the B2 NiAl played a role as a defect sink, the absorption of additional mobile irradiation interstitials worsened the distortion and finally drove the atoms to transform into two-dimensional defect structures like “twisted” planes. Considering the Moiré fringe region used to be a coherent B2 NiAl, the {100} plane would be of preference. For planes with more compact atom arrangements like {220} and {111}, they could result from interactions between precipitates and mobile radiation-induced dislocations. However, such metastable planes could only maintain a small size and would lose their structure with increasing areas and be transformed into {100} again, which was consistent with the dislocation results. In other words, the radiation-enhanced diffusion played a significant role in lattice recovery process by restoring the interstitial atoms while the low temperature inhibited perfect returns to the lattice sites.

### 3.4. Irradiation-Induced Dislocation Loops

**Figure 7:**
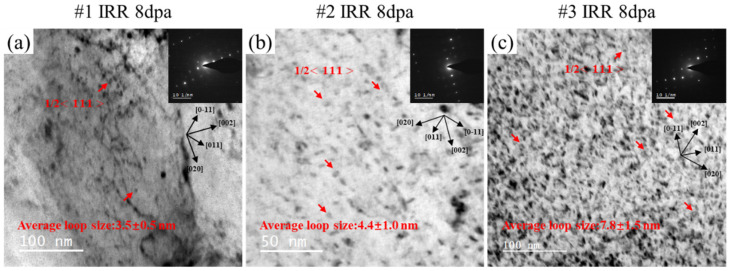
The two-beam kinematical BF images of three post-aging samples after irradiation. (**a**) #1; (**b**) #2; (**c**) #3.

A large number of dislocation loops were found in the two-beam kinematical bright-field (KMBF) TEM image after irradiation, as shown in Figure 7. Figure 7a corresponds to KMBF with g = 002 excited, viewed near the [001] axis of #1 after irradiation. The drawn crystal orientation was marked in the figure and the red arrow marked the a/2<111> dislocation loops. The same analysis method was carried out for the dislocation loop in Figure 7b,c. Generally, because of the low mobility of vacancies at room temperature and the preference for vacancies to aggregate into vacancy clusters, the observable dislocation loops are more likely to be of the interstitial type rather than of the vacancy type. The results are the same as expected, most of the dislocation loops in the irradiated Corrax are a/2<111> dislocation loops. The size of the irradiation-induced dislocation loops of #1, #2 and #3 were analyzed and results show that, under the same irradiation conditions, the average diameters of the dislocation loops in #1, #2 and #3 were 3.5 ± 0.5 nm, 4.4 ± 1.0 nm and 7.8 ± 1.5 while the number density was 6.11 × 10^20^, 1.17 × 10^21^ and 1.21 × 10^21^ m^−3^ nm, respectively. The size of the dislocation loops increased along with the larger-sized precipitates in the sample.

## 4. Conclusions

The present work investigated the stability of the B2 NiAl precipitate phase and the microstructure characteristics in PH 13-8 Mo steel. Irradiation experiments were performed on PH 13-8 Mo that underwent thermal aging at 723 K, 748 K and 773 K. The samples were irradiated with 400 keV Fe^+^ ions at room temperature with a cumulative irradiation damage dose of 8 dpa. The microstructural changes were quantitatively analyzed by TEM and EDS. Key findings are summarized as follows:

1. Before irradiation, controlled state samples thermally aged at 723 K, 748 K and 773 K for 20 h presented uniformly distributed B2 NiAl precipitates in the matrix with sizes of 2.5, 4.9 and 8.1 nm, respectively.
2. After irradiation with given conditions, the superlattice diffraction spots of B2 NiAl of smaller size disappeared while the larger ones mostly survived. No elemental diffusion was found by EDS, indicating radiation-induced precipitate disordering is the main reason for radiation dissolution. The variation in superlattices was attributed to the sink strength of the precipitates, and the recoil dissolution was controlled by the critical size 8.1 nm.
3. The Moiré fringes could be explained as {100}, {220} and {111} planes twisted with certain angles overlapped with the matrix in HRTEM images. The most observed defect planes were induced by the recoil dissolution of precipitates or interactions between precipitates and nearby mobile irradiation dislocations.
4. This work not only improves the theoretical understanding, of the radiation tolerance of PH 13–8 Mo steel in irradiated environments but also provides solidate experimental data to gain insights into intermetallic strengthened material designs in the future.

## Figures and Tables

**Table 1:** Nominal composition of PH 13–8 Mo (wt.% and at.%).

Element	C	Si	Mn	Cr	Ni	Mo	Al	Fe
wt.%	0.03	0.24	0.29	11.9	9.11	1.36	1.78	Bal.
at.%	0.13	0.47	0.29	12.52	8.49	0.78	3.61	Bal.

**Table 2:** Average size and number density of NiAl after heat treatment.

Number	Heat Treatment	Average Size (nm)	Number Density 10^22^/m^3^
#1	723 K, 20 h	2.5 ± 0.3	2.01 ± 0.161
#2	748 K, 20 h	4.9 ± 0.3	0.94 ± 0.015
#3	773 K, 20 h	8.1 ± 0.3	0.35 ± 0.004

**Table 3:** Solutions of Moiré patterns.

	*d_F/M_*_(110)_ (nm)	*d_m_* (nm)	*Φ* (°)	Solution#1			Solution#2		
				*d_NiAl_*_(*hkl*)_	*α* (°)	$\frac{d_{0(110)}^{2}}{d_{1(hkl)}^{2}}$	*d_NiAl_*_(*hkl*)_	*α* (°)	$\frac{d_{0(110)}^{2}}{d_{1(hkl)}^{2}}$
#1A	0.294295	0.507257	65.61	0.317820	34.389127	0.85744	0.218401	21.486198	1.815751
#1B	0.294295	0.578619	73.92	0.324969	30.000012	0.82013	0.197647	10.447733	2.217108
#2	0.292533	0.475533	53.92	0.400547	32.114138	0.533389	0.245788	Invalid	1.416547
#3	0.297648	0.632557	58.34	0.348979	28.125003	0.727454	0.22726	17.188734	1.715375

**Table 4:** Identified lattice planes.

Equivalent Results	Plane Family of *d*_1_	Twisted Angle (°)	Plane	Twisted Angle (°)
#1A	100	34	220	21
#1B	100	30	111	10
#2	100	30	-	-
#3	100	28	-	-

## Data Availability

The original contributions presented in this study are included in the article. Further inquiries can be directed to the corresponding author.

## Abbreviations

The following abbreviations are used in this manuscript:

SAED	Selected Area Electron Diffraction
STEM	Scanning Transmission Electron Microscopy
EDS	Energy Dispersive Spectroscopy
ITER	International Thermonuclear Experimental Reactor
TEM	Transmission Electron Microscope
APT	Atom Probe Tomography
HRTEM	High Resolution Transmission electron microscope
DF	Dark Field
BF	Bright Field
CBED	Convergent Beam Electron Diffraction
FFT	Fast Fourier Transform
IFFT	Inverse Fast Fourier Transform
DM	Digital Micrograph
MD	Molecular Dynamic
PKA	Primary Knock Atom
BCC	Body-Centered Cubic
